# Personalized Prediction of Lifetime Benefits with Statin Therapy for Asymptomatic Individuals: A Modeling Study

**DOI:** 10.1371/journal.pmed.1001361

**Published:** 2012-12-27

**Authors:** Bart S. Ferket, Bob J. H. van Kempen, Jan Heeringa, Sandra Spronk, Kirsten E. Fleischmann, Rogier L. G. Nijhuis, Albert Hofman, Ewout W. Steyerberg, M. G. Myriam Hunink

**Affiliations:** 1 Department of Epidemiology, Erasmus MC, Rotterdam, the Netherlands; 2 Department of Radiology, Erasmus MC, Rotterdam, the Netherlands; 3 Department of Medicine, University of California, San Francisco, California, United States of America; 4 Department of Cardiology, Ziekenhuisgroep Twente, Hengelo, the Netherlands; 5 Department of Public Health, Erasmus MC, Rotterdam, the Netherlands; 6 Department of Health Policy and Management, Harvard School of Public Health, Boston, Massachusetts, United States of America; The George Institute for International Health, Australia

## Abstract

**Background:**

Physicians need to inform asymptomatic individuals about personalized outcomes of statin therapy for primary prevention of cardiovascular disease (CVD). However, current prediction models focus on short-term outcomes and ignore the competing risk of death due to other causes. We aimed to predict the potential lifetime benefits with statin therapy, taking into account competing risks.

**Methods and Findings:**

A microsimulation model based on 5-y follow-up data from the Rotterdam Study, a population-based cohort of individuals aged 55 y and older living in the Ommoord district of Rotterdam, the Netherlands, was used to estimate lifetime outcomes with and without statin therapy. The model was validated in-sample using 10-y follow-up data. We used baseline variables and model output to construct (1) a web-based calculator for gains in total and CVD-free life expectancy and (2) color charts for comparing these gains to the Systematic Coronary Risk Evaluation (SCORE) charts. In 2,428 participants (mean age 67.7 y, 35.5% men), statin therapy increased total life expectancy by 0.3 y (SD 0.2) and CVD-free life expectancy by 0.7 y (SD 0.4). Age, sex, smoking, blood pressure, hypertension, lipids, diabetes, glucose, body mass index, waist-to-hip ratio, and creatinine were included in the calculator. Gains in total and CVD-free life expectancy increased with blood pressure, unfavorable lipid levels, and body mass index after multivariable adjustment. Gains decreased considerably with advancing age, while SCORE 10-y CVD mortality risk increased with age. Twenty-five percent of participants with a low SCORE risk achieved equal or larger gains in CVD-free life expectancy than the median gain in participants with a high SCORE risk.

**Conclusions:**

We developed tools to predict personalized increases in total and CVD-free life expectancy with statin therapy. The predicted gains we found are small. If the underlying model is validated in an independent cohort, the tools may be useful in discussing with patients their individual outcomes with statin therapy.

*Please see later in the article for the Editors' Summary*

## Abstract 2

In a modeling study conducted by Myriam Hunink and colleagues, a population-based cohort from Rotterdam is used to predict the possible lifetime benefits of statin therapy, on a personalized basis.

## Editors' Summary

**Background:**

Cardiovascular disease (CVD) affects the heart and/or the blood vessels and is a major cause of illness and death worldwide. In the US, for example, coronary heart disease—a CVD in which narrowing of the heart's blood vessels by fatty deposits slows the blood supply to the heart and may eventually cause a heart attack—is the leading cause of death, and stroke—a CVD in which the brain's blood supply is interrupted—is the fourth leading cause of death. Established risk factors for CVD include smoking, high blood pressure, obesity, and high blood levels of a fat called low-density lipoprotein (“bad cholesterol”). Because many of these risk factors can be modified by lifestyle changes and by drugs, CVD can be prevented. Thus, physicians can assess a healthy individual's risk of developing CVD using a CVD prediction model (equations that take into account the CVD risk factors to which the individual is exposed) and can then recommend lifestyle changes and medications to reduce that individual's CVD risk.

**Why Was This Study Done?:**

Current guidelines recommend that asymptomatic (healthy) individuals whose likely CVD risk is high should be encouraged to take statins—cholesterol-lowering drugs—as a preventative measure. Statins help to prevent CVD in healthy people with a high predicted risk of CVD, but, like all medicines, they have some unwanted side effects, so it is important that physicians can communicate both the benefits and drawbacks of statins to their patients in a way that allows them to make an informed decision about taking these drugs. Telling a patient that statins will reduce his or her short-term risk of CVD is not always helpful—patients really need to know the potential lifetime benefits of statin therapy. That is, they need to know how much longer they might live if they take statins. Here, the researchers use a mathematical model to predict the personalized lifetime benefits (increased total and CVD-free life expectancy) of statin therapy for individuals without a history of CVD.

**What Did the Researchers Do and Find?:**

The researchers used the Rotterdam Ischemic Heart Disease & Stroke Computer Simulation (RISC) model, which simulates the life courses of individuals through six health states, from well through to CVD or non-CVD death, to estimate lifetime outcomes with and without statin therapy in a population of healthy elderly individuals. They then used these outcomes and information on baseline risk factors to develop a web-based calculator suitable for personalized prediction of the lifetime benefits of statins in routine clinical practice. The model estimated that statin therapy increases average life expectancy in the study population by 0.3 years and average CVD-free life expectancy by 0.7 years. The gains in total and CVD-free life expectancy associated with statin therapy increased with blood pressure, unfavorable cholesterol levels, and body mass index (an indicator of body fat) but decreased with age. Notably, the web-based calculator predicted that some individuals with a low ten-year CVD risk might achieve a similar or larger gain in CVD-free life expectancy with statin therapy than some individuals with a high ten-year risk. So, for example, both a 55-year-old non-smoking woman with a ten-year CVD mortality risk of 2% (a two in a hundred chance of dying of CVD within ten years) and a 65-year-old male smoker with a ten-year CVD mortality risk of 15% might both gain one year of CVD-free life expectancy with statin therapy.

**What Do These Findings Mean?:**

These findings suggest that statin therapy can lead on average to small gains in total life expectancy and slightly larger gains in CVD-free life expectancy among healthy individuals, and show that life expectancy benefits can be predicted using an individual's risk factor profile. The accuracy and generalizability of these findings is limited by the assumptions included in the model (in particular, the model did not allow for the known side effects of statin therapy) and by the data fed into it—importantly, the risk prediction model needs to be validated using an independent dataset. If future research confirms the findings of this study, the researchers' web-based calculator could provide complementary information to the currently recommended ten-year CVD mortality risk assessment. Whether communication of personalized outcomes will ultimately result in better clinical outcomes remains to be seen, however, because patients may be less likely to choose statin therapy when provided with more information about its likely benefits.

**Additional Information:**

Please access these websites via the online version of this summary at http://dx.doi.org/10.1371/journal.pmed.1001361.

- The web-based calculator for personalized prediction of lifetime benefits with statin therapy is available (after agreement to software license)
- The American Heart Association provides information about many types of cardiovascular disease for patients, carers, and professionals, including information about drug therapy for cholesterol and a heart attack risk calculator
- The UK National Health Service Choices website provides information about cardiovascular disease and about statins
- Information is available from the British Heart Foundation on heart disease and keeping the heart healthy; information is also available on statins, including personal stories about deciding to take statins
- The US National Heart Lung and Blood Institute provides information on a wide range of cardiovascular diseases
- The European Society of Cardiology's cardiovascular disease risk assessment model (SCORE) is available
- MedlinePlus provides links to many other sources of information on heart diseases, vascular diseases, stroke, and statins (in English and Spanish)

## Introduction

Current guidelines recommend that asymptomatic individuals at high cardiovascular disease (CVD) risk be identified for statin therapy. For this purpose, risk assessment is performed using prediction models estimating short-term, i.e., 5- to 10-y CVD risk [1],[2]. The higher the predicted CVD risk, the stronger the recommendation is to initiate statin therapy. This reasoning is based on solid evidence demonstrating a CVD-risk-reducing effect for statins [3],[4], with an expected larger absolute benefit as CVD risk increases [5]. For shared decision-making, physicians need to communicate to the patient personalized information about the outcomes of statin therapy [6]. Whether the magnitude of the expected benefit would outweigh the disadvantages of statin therapy (e.g., side effects, the disutility of taking a pill every day) can be discussed with the individual in order to reach agreement on initiation of the drug therapy.

Using the currently available short-term CVD prediction models for estimating treatment benefits has limitations. First, statin therapy is generally continued over the remainder of the course of the lifetime, and information for decision-making should reflect the expected long-term benefit [7]. Second, short-term risk reductions are generally small and difficult to interpret by lay people [8]. Third, competing risk of death due to causes other than CVD is generally not taken into account. Especially in frail individuals, who are also at high risk of dying from other causes, ignoring the competing risk of non-CVD death leads to overestimation of CVD risk and thus overestimation of the treatment benefit [9]. Decision models have the ability to extrapolate short-term follow-up data to a lifetime horizon while taking into account competing risks of death. Results can be expressed on a time scale, as gains or losses in (CVD-free) life expectancy. Life expectancy measures have the advantage that the aggregated treatment benefits over the full life span can be represented by a single value. This could provide information complementary to the conventional communication of risk reduction, which is limited to the use of fixed time points [10]. Presenting data in various different ways can be helpful for assessing the certainty about therapy choices and could improve the quality of decision-making [11].

Our aim was to predict personalized lifetime benefits with statin therapy for prevention of CVD in asymptomatic individuals without a history of CVD.

## Methods

### The Decision Model

We used a previously developed microsimulation state-transition model, the Rotterdam Ischemic Heart Disease & Stroke Computer Simulation Model (RISC model), which was built in TreeAge (version Data Professional, release 10, TreeAge Software) [12]. The RISC model was developed using 7-y follow-up data from 3,501 participants of the Rotterdam Study, a population-based cohort study of individuals aged 55 y and older living in the Ommoord district of Rotterdam, the Netherlands, followed from 1990 onwards. Only participants with complete data on the baseline risk factors were used in the development of the RISC model [13]. Instead of using 7-y hazard rates, more stable 5-y hazard rates were used for extrapolation to a lifetime horizon in order to evaluate the lifetime effects of CVD preventive strategies.

**Figure 1:**
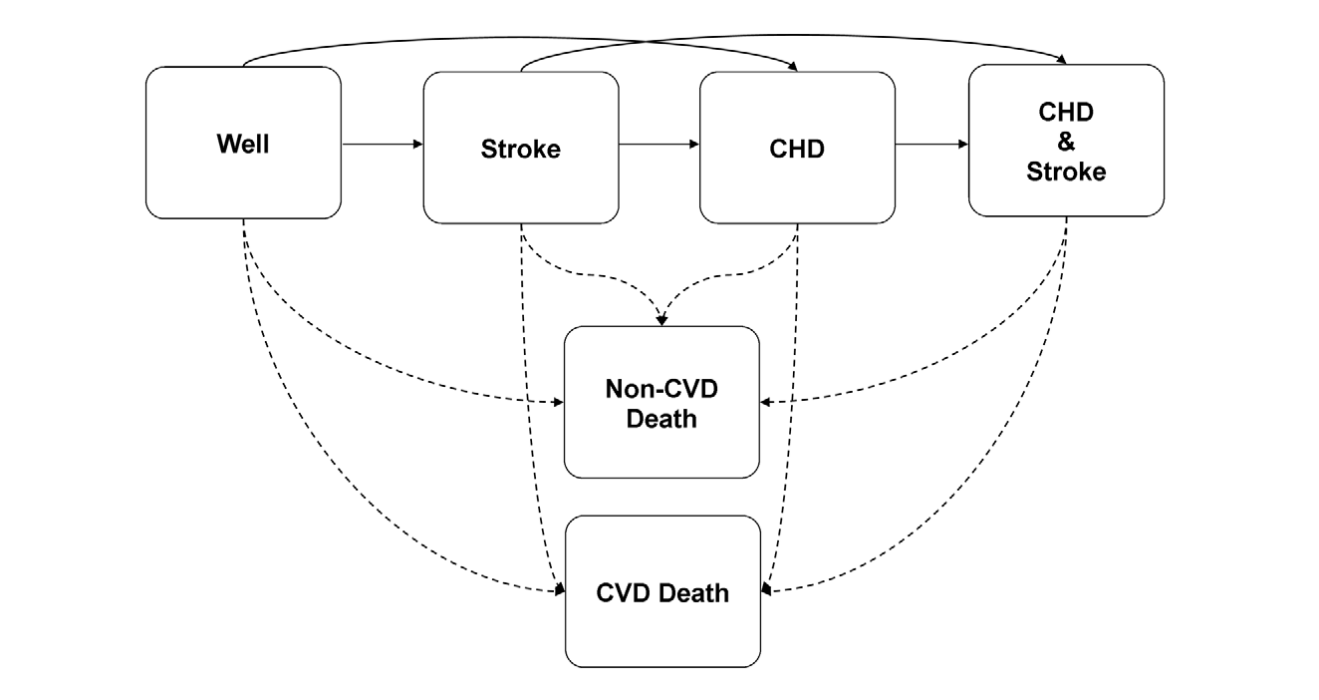
Schematic representation of the RISC model.

In the model, life courses of participants were simulated using six health states: well, after non-fatal coronary heart disease (CHD), after non-fatal stroke, after non-fatal CHD and non-fatal stroke, cardiovascular death, and non-cardiovascular death (see Figure 1). CHD was defined as having experienced acute myocardial infarction (International Classification of Diseases, 10th edition [ICD-10] code I21) or having undergone percutaneous transluminal coronary angioplasty or coronary artery bypass grafting. Stroke was limited to non-hemorrhagic and unspecified strokes (ICD-10 codes I63 and I64) in order to be able to model the adverse bleeding risk of preventive interventions such as aspirin therapy separately. Cardiovascular death was defined as mortality due to hypertensive diseases (ICD-10 codes I10–I15), ischemic heart disease (ICD-10 codes I20–I25), sudden cardiac death (ICD-10 codes I46 and I49), congestive heart failure (ICD-10 code I50), cerebrovascular disease (ICD-10 codes I60–I67), other arterial disease (ICD-10 codes I70–I79), or sudden death (ICD-10 code R96). Non-cardiovascular death was defined as mortality due to all other causes (all other ICD-10 codes). During 5 y of follow-up, 176 CHD events, 127 stroke events, 165 CVD deaths, and 264 non-CVD deaths occurred in the population of 3,501 participants used to develop the model. Transitions between health states were individualized using multivariable Cox regression models, while adjusting for competing risk. Consequently, the “one-cycle cumulative incidence” for each event was calculated as the ratio of the cumulative hazard of the event of interest, censored for all other events, to the cumulative hazard of any event, multiplied by the probability of any event. If constant hazards are assumed within each cycle, the overall cumulative incidences will be estimated correctly [14]. The Cox regression models were fitted in 100 bootstrapped datasets to take into account the parameter uncertainty of hazard ratios. Each simulated individual entered the model starting in the well state, with his or her baseline risk profile. Secular trends in risk factor levels were modeled across the age span using cross-sectional analyses of baseline data. The individual's risk profile at baseline and (if alive) the updated risk profile at the beginning of each simulated subsequent 5-y period was used as input for the Cox regression equations. In addition, the Cox regression equations included age–risk factor interactions. Two life course scenarios were modeled: “with statin therapy” versus “without statin therapy.” A cycle length of 1 y without discounting was applied to provide an “actual” life expectancy (for more information about the RISC model, see Text S1).

### Model Validity

The RISC model was constructed with extrapolation of 5-y predictions based on 7-y follow-up data of 3,501 participants. However, at the time point of this analysis, we had access to data with a mean follow-up duration of 11.8 y including 367 CHD events, 343 stroke events, 494 CVD deaths, and 846 non-CVD deaths. Therefore, we were able to evaluate the validity of extrapolation to a longer term by comparing simulated and observed cumulative incidences at 5 and 10 y of follow-up. We modeled the life courses of the 3,501 Rotterdam Study participants. To assess parameter uncertainty, we calculated 95% confidence intervals (95% CIs) by consecutively sampling beta coefficient estimates from the Cox regression analyses performed in the 100 bootstrapped datasets. Observed cumulative incidences and 95% CIs were calculated, taking into account competing death risks and loss to follow-up, using the R cuminc function available from the mstate package. To assess model discrimination, we calculated the Harrell's C-statistic [15] for 10-y CHD events, stroke events, CVD mortality, and non-CVD mortality. We adjusted the C-statistic for competing risk by setting the censoring time to “infinity” (i.e., the maximum follow-up time of 10 y+1) for those who died of causes other than the event of interest [9].

In addition, we compared the 10-y CVD mortality risk from the RISC model with the European Society of Cardiology Systematic Coronary Risk Evaluation (SCORE) charts. Because uncertainty exists about which SCORE charts to use for Dutch individuals [16], we compared 10-y CVD mortality risk to the three available versions: high-risk region, low-risk region, and Dutch recalibrated SCORE charts. SCORE 10-y CVD mortality risks were calculated using the equations provided by Conroy et al. [17] and van Dis et al. [16]. For calculation of the RISC model's 10-y CVD mortality risk, we included death by CVD other than stroke and CHD. The RISC model's average 10-y CVD mortality risk estimations and the predictions from each SCORE equation were plotted by tenths of predicted 10-y CVD mortality from the RISC model. This was done for only a subset of 1,047 asymptomatic participants younger than 65 y, meeting the population criteria for which the SCORE equations are applicable [17]. The 95% CIs of estimates from the RISC model were calculated by sampling from the 100 beta coefficient bootstrap replicates as previously described; the 95% CIs of SCORE predictions were estimated using non-parametric bootstrapping of the data in each tenth.

### Statin Therapy Efficacy

The effect of statin therapy was modeled on the occurrence of first CHD and stroke events in 2,428 participants who did not use statin therapy at baseline and were free of CVD (defined as myocardial infarction, transient ischemic attack, stroke diagnosed by a physician, and/or a self-reported history of coronary artery bypass grafting, percutaneous transluminal coronary angioplasty, or carotid surgery), angina pectoris, intermittent claudication, and atrial fibrillation. We conservatively assumed that there was no statin effect on direct transitions from the well state to the cardiovascular death state, and that the effect of statins was solely effectuated through their effect on CHD and stroke events. We did not model additional therapy effects after occurrence of CVD and did not consider the negligible fatal adverse effects of statin therapy [18]. The odds ratios for first CHD and stroke events were derived from a recent meta-analysis (see Table S1) [3]. This meta-analysis provides effect estimates for statins at doses that are generally recommended for primary prevention. We assumed that adherence to statin therapy was adequately captured in the statin effect, as observed in trials with an intention-to-treat analysis. Because benefits are known to be significant within the first year of treatment [19], we assumed that the full extent of the statin effect was achieved within 1 y. In addition, we kept odds ratios constant over all ages and risk factor levels [3],[20].

### Personalized Prediction of Lifetime Benefits

We ran the RISC model for the 2,428 participants under the scenarios with and without statin therapy. To take into account parameter uncertainty of the Cox regression beta coefficients underlying the state transition probabilities, 100 linked sets of coefficients were derived using bootstrapping. Odds ratios with statin therapy for first CHD and stroke events were randomly sampled using log-normal distributions based on the reported 95% CIs. To limit the stochastic error in event occurrences, we used 200 random walks per parameter set. Thus, the RISC model output consisted of the average lifetime outcomes from 20,000 runs per participant (100 parameter sets×200 random walks) under the two scenarios (with statin therapy versus without statin therapy). The uncertainty in the predictions was addressed by running the RISC model while aggregating at the parameter level. To show this parameter uncertainty, we present average outcomes with 95% CIs. Heterogeneity was addressed by running the RISC model while aggregating at the individual level (Rotterdam Study participants); the standard deviations (SDs) presented represent the variation in outcomes across individuals.

Because it is infeasible to run the complicated RISC model for use in clinical practice, we developed easily programmable equations that predict the RISC model's output using the baseline risk profile of the individual. We used the data generated by the RISC model while aggregating at the individual level, as described above. Depending on the outcome chosen, linear and generalized linear models with repeated measure statements were used for constructing these equations. Our primary outcomes were total life expectancy and CVD-free (CHD/stroke-free) life expectancy. In addition, we predicted the lifetime risk of developing a first CHD or stroke event (either fatal or non-fatal), lifetime CHD/stroke mortality risk, and lifetime total CVD mortality risk. We selected the following candidate predictors: age, sex, current smoking, systolic and diastolic blood pressure, hypertension (defined as either reporting use of antihypertensive medication and/or a systolic blood pressure ≥160 mm Hg or a diastolic blood pressure ≥95 mm Hg at baseline), total cholesterol, high-density lipoprotein (HDL) cholesterol, diabetes mellitus (defined as either reporting use of antidiabetic medication and/or a random or post-load serum glucose level ≥11.0 mmol/l at baseline), serum glucose, body mass index, waist-to-hip ratio, and serum creatinine. We chose these variables because they can be reliably and easily obtained during an office-based health check. Interactions with statin therapy, age, and sex were tested. Continuous variables were entered as linear and quadratic terms. Final models were selected based on the Akaike's information criterion, which calculates the log-likelihood penalized for the number of parameters used. All analyses were performed using R version 2.12.2 (R Foundation for Statistical Computing, http://www.r-project.org/). For details on statistical analyses see Text S1.

The predictions of the RISC model have not been independently validated and are thus not ready for clinical use. However, to facilitate validation, we developed a web-based calculator using the Cleveland Clinic Risk Calculator Constructor (http://rcc.simpal.com/). The calculator is available at http://www.erasmusmc.nl/clinical-epidemiology/patientcare/. (Note: as the calculator is constructed using software hosted by the Cleveland Clinic, users are asked to agree to the software license of this organization upon first use.) To illustrate the output of the web-based calculator, we contrasted the expected lifetime benefits (expressed in total life expectancy and CHD/stroke-free life expectancy) with statin therapy to 10-y total CVD mortality risks for four different risk profiles.

In order to compare gains in total and CHD/stroke-free life expectancy with office-based assessment of 10-y total CVD mortality risk, as recommended in the European Society of Cardiology 2007 guidelines, we constructed color charts similar to the SCORE risk charts. To show the distribution of the simulated gains in total and CHD/stroke-free life expectancy according to SCORE risk estimations we drew scatter plots for the asymptomatic population younger than 65 y.

### Ethics Statement and Data Access

The Rotterdam Study has been approved by the institutional review board (Medical Ethics Committee) of the Erasmus Medical Center and by the review board of the Netherlands Ministry of Health, Welfare, and Sports. The approval has been renewed every 5 y. The steering committee of the Rotterdam Study does not allow free sharing of data. Currently, Rotterdam Study data are shared only within collaborative research projects. Therefore, the data needed for constructing the web-based calculator unfortunately cannot be made available for altering to different scenarios.

## Results

### Model Validity

At year 5, the observed (95% CI) versus simulated (95% CI) incidences of CHD, stroke, CVD, and non-CVD mortality were 5.0% (4.3–5.8) versus 4.7% (4.2–5.4), 3.6% (3.0–4.3) versus 3.2% (2.7–3.8), 4.7% (4.0–5.4) versus 4.8% (3.6–6.1), and 7.6% (6.7–8.5) versus 8.1% (7.1–9.2), respectively. At year 10, these percentages were 8.5% (7.6–9.5) versus 8.9% (7.9–10.0), 7.6% (6.7–8.5) versus 6.9% (5.9–8.1), 10.9% (9.9–12.0) versus 10.9% (8.6–13.6), and 17.7% (16.5–19.0) versus 17.9% (16.1–20.0). The C-statistic (95% CI) for CHD was 0.73 (0.70–0.76), for stroke 0.67 (0.64–0.70), for CVD mortality 0.80 (0.78–0.82), and for non-CVD mortality 0.74 (0.72–0.76).

In the 1,047 participants younger than 65 y, the low-risk region SCORE equation provided 10-y total CVD mortality estimations that were most similar to the RISC model output (see Figure S1). The other two SCORE equations overestimated 10-y total CVD mortality risk as compared to the RISC model, particularly in the upper two deciles of SCORE risk estimations (see Figures S2 and S3).

### Population Results

The baseline characteristics of the study population are summarized in Table 1. In the 2,428 participants (mean age 67.7, SD 8.1, 35.5% men), the average total life expectancy without statin therapy was 18.3 (SD 6.5) y. The average remaining life expectancy for females (males) at the age of 60 y was 25.5 (20.4) y, at 65 y was 21.4 (16.7) y, and at 80 y was 10.5 (7.0) y. Values were less favorable in the original Rotterdam Study cohort including symptomatic individuals (*n* = 3,501): 25.3 (19.8) y, 21.1 (16.1) y, and 10.2 (6.6) y, respectively. Average CHD/stroke-free life expectancy in the asymptomatic study population was 16.0 (SD 5.8) y; for females (males), values were 21.8 (16.4) y at the age of 60 y, 18.4 (13.5) y at 65 y, and 9.6 (5.6) y at 80 y.

**Table 1:** Characteristics of 2,428 participants aged 55 y and older, free of cardiovascular disease and symptoms at baseline.

Characteristics	RISC Model Study Population
**Age (years)**	67.7 (8.1)
2.5th–97.5th range	55–85
**Male sex—number (percent)**	863 (35.5)
**Current cigarette smoking—number (percent)**	582 (24.0)
**Blood pressure (mm Hg)**	
Systolic	139.2 (22.4)
2.5th–97.5th range	100–186
Diastolic	74.7 (11.6)
2.5th–97.5th range	53–98
**Hypertension—number (percent)**	768 (31.6)
**Serum cholesterol (mmol/l)**	6.7 (1.3)
2.5th–97.5th range	4.5–9.2
**Serum HDL cholesterol (mmol/l)**	1.4 (0.4)
2.5th–97.5th range	0.8–2.2
**Diabetes mellitus—number (percent)**	215 (8.9)
**Serum glucose (mmol/l)**	6.8 (2.5)
2.5th–97.5th range	4.3–13.6
**Body mass index (kg/m^2^)**	26.2 (4.3)
2.5th–97.5th range	20.1–34.3
**Waist-to-hip ratio**	0.90 (0.09)
2.5th–97.5th range	0.73–1.08
**Serum creatinine (µmol/l)**	80.6 (15.8)
2.5th–97.5th range	58–110

Data are mean (SD) unless otherwise indicated. Hypertension is defined as either reporting use of antihypertensive medication or having a systolic blood pressure ≥160 mm Hg or a diastolic blood pressure ≥95 mm Hg. Diabetes mellitus is defined as either reporting use of antidiabetic medication or having a serum glucose level ≥11.0 mmol/l.

Statin therapy resulted in an average gain in life expectancy of 0.3 (95% CI 0.2–0.3) y, with a range of 0.0 to 2.0 y. The gain in CHD/stroke-free life expectancy with statin therapy was 0.7 (95% CI 0.5–1.0) y, with a range of 0.1 to 2.8 y. The absolute risk reduction in CVD incidence with statin therapy was larger than the decrease in CVD mortality: 6.6% (95% CI 4.5–8.5) versus 3.0% (95% CI 2.0–3.9). The competing other CVD and non-CVD lifetime mortality risks increased with statin therapy, by 0.9% (95% CI 0.3–1.7) and 2.1% (95% CI 1.3–3.0), respectively. The effects of statin therapy on the various outcomes are summarized in Table 2. Both the heterogeneity (SDs and ranges) and the parameter uncertainty (95% CIs) of gains with statin therapy are shown.

**Table 2:** Predicted outcomes and changes with statin therapy for the study population (*n* = 2,428) aged 55 y and older, free of cardiovascular disease and symptoms at baseline.

Outcome	Mean Baseline Value (SD)	Mean Absolute Change (SD)	Minimum; Maximum Absolute Change	95% CI Absolute Change
Total life expectancy (years)	18.3 (6.5)	+0.3 (0.2)	0.0; +2.0	+0.2; +0.3
CHD/stroke-free life expectancy (years)	16.0 (5.8)	+0.7 (0.4)	+0.1; +2.8	+0.5; +1.0
CHD/stroke incidence (percent)	33.2 (10.6)	−6.6 (1.7)	−11.0; −2.8	−8.5; −4.5
CHD/stroke mortality (percent)	12.8 (5.3)	−3.0 (1.2)	−11.5; −0.9	−3.9; −2.0
Other CVD mortality (percent)	26.0 (8.7)	+0.9 (0.7)	−0.8; +6.8	+0.3; +1.7
Non-CVD mortality (percent)	61.3 (10.9)	+2.1 (0.9)	+0.1; +7.7	+1.3; +3.0

The means, SDs, and ranges are presented to reflect the heterogeneity in the predicted outcomes, and 95% CIs to reflect the parameter uncertainty.

### Personalized Prediction of Lifetime Benefits

For the use of the web-based calculator (http://www.erasmusmc.nl/clinical-epidemiology/patientcare/), information on 13 predictors is required: age, sex, smoking, sytolic blood pressure (mm Hg), diastolic blood pressure (mm Hg), hypertension, total cholesterol (mmol/l), HDL cholesterol (mmol/l), diabetes mellitus, serum glucose (mmol/l), body mass index (kg/m^2^), waist-to-hip ratio, and serum creatinine (µmol/l). Ranges for possible values of continuous predictors were based on the 2.5th and 97.5th centiles of these variables in the 2,428 participants (see Table 1). Higher systolic blood pressure, higher total cholesterol, lower HDL cholesterol, and larger body mass index considerably increased gains in total and CHD/stroke-free life expectancy with statin therapy, adjusted for the other co-variables. Increasing age, however, most notably decreased these gains. Diabetes mellitus also slightly decreased these gains. Effects of the other predictors on changes in total and CHD/stroke-free life expectancy were generally small.

Table 3 presents the 10-y total CVD mortality risks and lifetime outcomes with and without statin therapy for selected risk profiles. Participants with a low 10-y CVD risk can achieve a similar or larger gain in (CHD/stroke-free) life years with statin therapy than participants with a high 10-y risk. For example, a 55-y-old non-smoking female at a 10-y risk of 2% could achieve a similar gain in (CHD/stroke-free) life expectancy with statin therapy as a 65-y-old smoking male at a 10-y risk of 15% (see risk profiles 1 and 2 in Table 3). A 55-y-old non-smoking male with hypercholesterolemia and hypertension at a 3% 10-y risk can achieve a larger gain in (CHD/stroke-free) life years with statin therapy than a 75-y-old smoking male with hypertension and diabetes at a 21% 10-y risk (see profiles 3 and 4 in Table 3).

**Table 3:** Changes (Δ) in total life expectancy and CHD/stroke-free life expectancy with statin therapy, compared with predicted 10-y total CVD mortality risk for different risk factor profiles.

Risk Profile	Total Life Expectancy in Years		CHD/Stroke-Free Life Expectancy in Years		10-Y Total CVD Mortality
	No Statin	Δ^a^	No Statin	Δ^b^	
55-y-old non-smoking ♀, blood pressure 140/80 mm Hg, hypertension +, total cholesterol 6.0 mmol/l, HDL cholesterol 1.5 mmol/l, diabetes −, glucose 6.0 mmol/l, BMI 25.0, WHR 0.80, creatinine 80 µmol/l	28.9	+0.3	24.9	+1.0	2%
65-y-old smoking ♂, blood pressure 130/70 mm Hg, hypertension +, total cholesterol 7.0 mmol/l, HDL cholesterol 1.0 mmol/l, diabetes +, glucose 6.0 mmol/l, BMI 30.0, WHR 1.06, creatinine 90 µmol/l	13.1	+0.4	9.7	+1.0	15%
55-y-old non-smoking ♂, blood pressure 140/75 mm Hg, hypertension +, total cholesterol 7.0 mmol/l, HDL 1.3 mmol/l, diabetes −, glucose 6.5 mmol/l, BMI 27.0, WHR 1.00, creatinine 80 µmol/l	23.9	+0.4	18.7	+1.2	3%
75-y-old smoking ♂, blood pressure 120/80 mm Hg, hypertension +, total cholesterol 4.5 mmol/l, HDL 1.0 mmol/l, diabetes +, glucose 6.0 mmol/l, BMI 21.0, WHR 1.00, creatinine 90 µmol/l	6.5	+0.1	6.1	+0.1	21%

Hypertension is defined as either reporting use of antihypertensive medication or having a systolic blood pressure ≥160 mm Hg or a diastolic blood pressure ≥95 mm Hg; diabetes is defined as either reporting use of antidiabetic medication or having a serum glucose level ≥11.0 mmol/l. Predictions for lifetime CHD/stroke incidence, CHD/stroke mortality, and total CVD mortality for these risk profiles, are shown in the Table S2.

Conventional conversion factors: to convert HDL and total cholesterol to milligrams per deciliter, divide by 0.0259; creatinine to milligrams per deciliter, divide by 88.4; glucose to milligrams per deciliter, divide by 0.0555.

a The gain in total life expectancy in years can be computed as follows: 0.2632−0.0077×age (in years)+0.0138×[1 if male sex, 0 if not]−0.0115×[1 if current cigarette smoker, 0 if not]+0.0023×systolic blood pressure (in mm Hg)−0.0018×diastolic blood pressure (in mm Hg)+0.0479×[1 if hypertension, 0 if not]+0.0548×total cholesterol (in mmol/l)−0.1448×HDL cholesterol (in mmol/l)−0.0218×[1 if diabetes mellitus, 0 if not]+0.0086×serum glucose (in mmol/l)+0.0099×body mass index (in kg/m^2^)−0.3989×waist-to-hip ratio+0.0025×serum creatinine (in µmol/l).

b The gain in CHD/stroke-life expectancy in years can be computed as follows: 1.8854−0.0330×age (in years)+0.0470×[1 if male sex, 0 if not]+0.0049×systolic blood pressure (in mm Hg)−0.0040×diastolic blood pressure (in mm Hg)+0.1157×total cholesterol (in mmol/l)−0.3605×HDL cholesterol (in mmol/l)−0.0899×[1 if diabetes mellitus, 0 if not]+0.0049×serum glucose (in mmol/l)+0.0175×body mass index (in kg/m^2^)−0.2915×waist-to-hip ratio+0.0023×serum creatinine (in µmol/l).

BMI, body mass index; WHR, waist-to-hip ratio.

**Figure 2:**
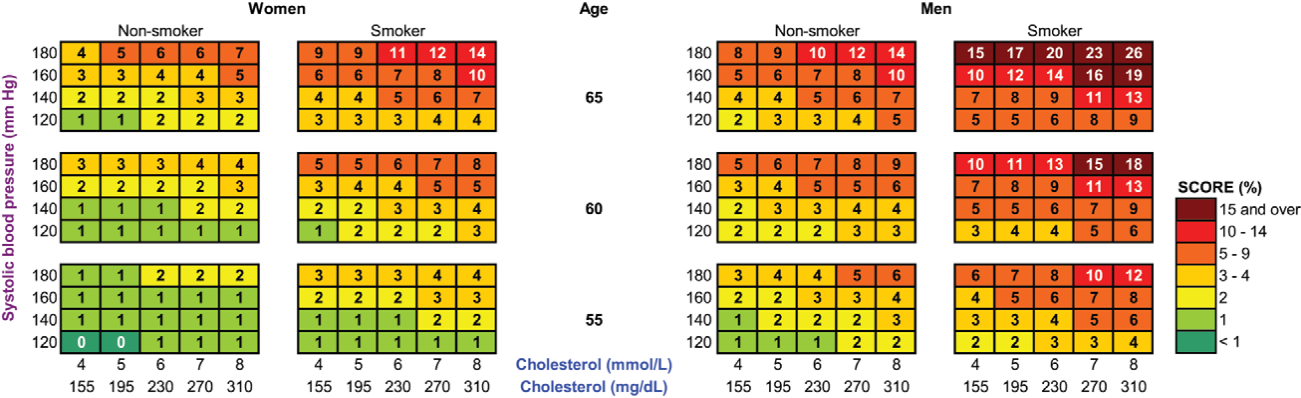
Ten-year total CVD mortality risk (percent) predicted by SCORE European low-risk charts. Adapted with permission from the European Society of Cardiology. Copyright: © 2007 Oxford University Press. Note that these charts demonstrate that the 10-y total CVD mortality risk is highest for elderly smoking individuals with otherwise high risk factor levels, suggesting that these individuals would benefit most from statin therapy.

**Figure 3:**
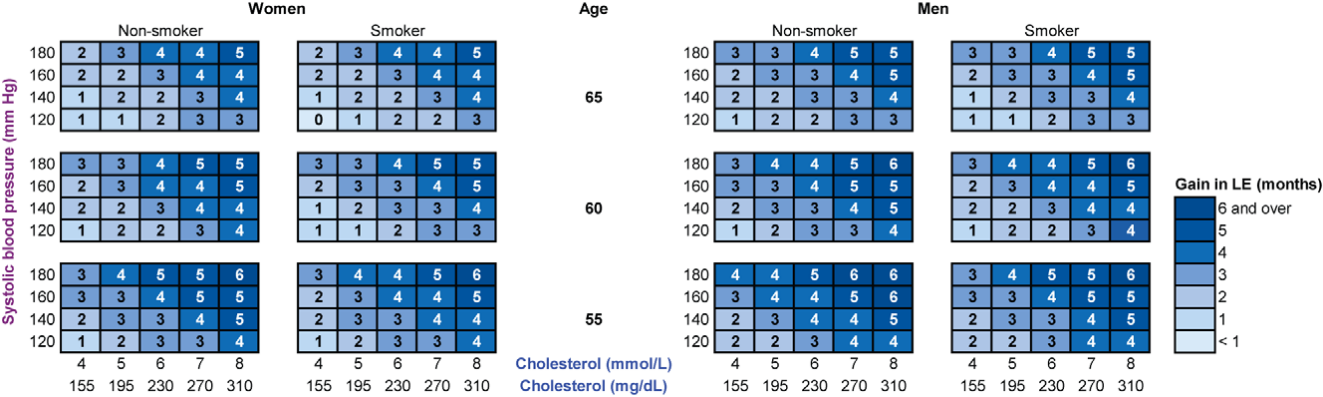
The gain in life expectancy (in months) with statin therapy, calculated with the RISC model. Note that these charts demonstrate that life expectancy (LE) gained with statin therapy is highest for young non-smoking individuals with otherwise high risk factor levels.

**Figure 4:**
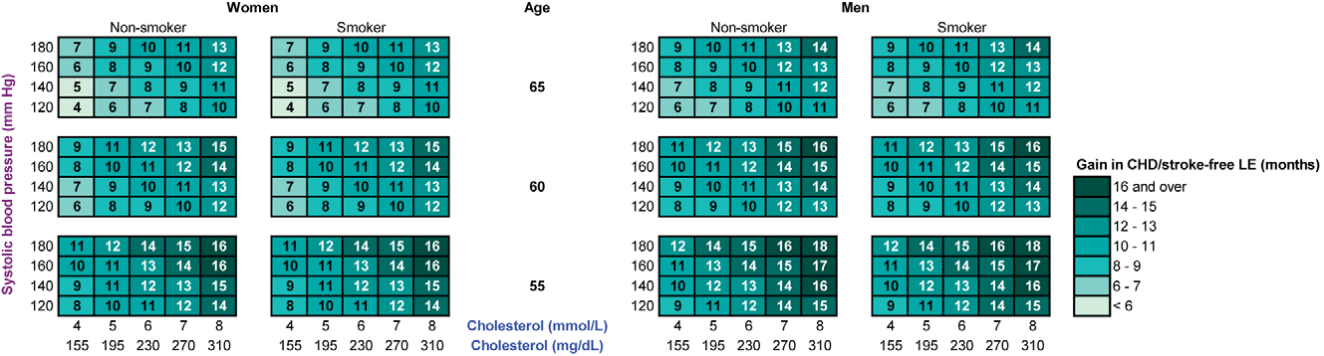
The gain in CHD/stroke-free life expectancy (in months) with statin therapy, calculated with the RISC model. Note that these charts demonstrate that CHD/stroke-free life expectancy (LE) gained with statin therapy is highest for young individuals with otherwise high risk factor levels.

**Figure 5:**
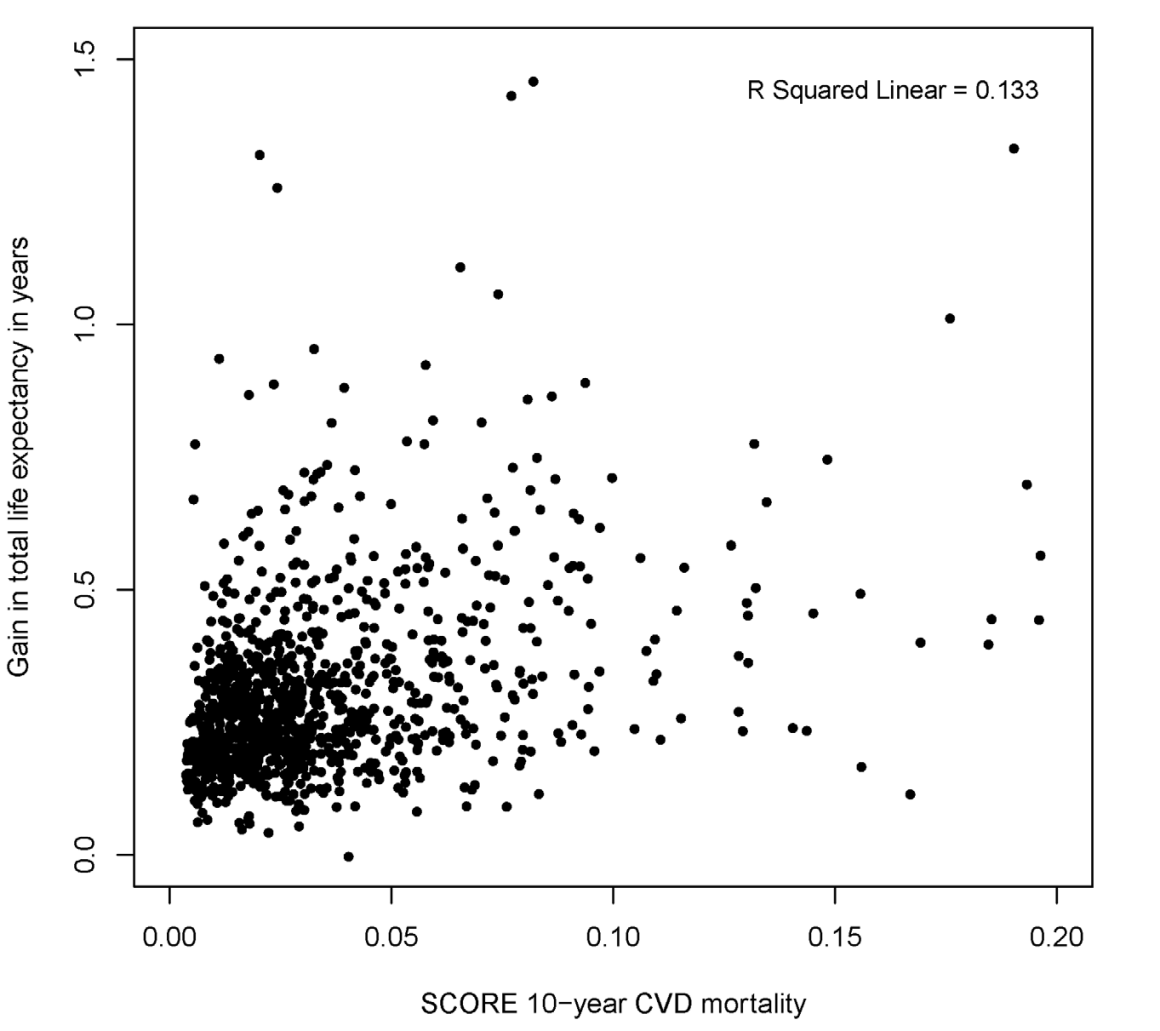
Distribution of gains in total life expectancy according to SCORE 10-y total CVD mortality risk (percent). Note that many individuals with a low SCORE 10-y CVD mortality risk achieved similar or higher gains than those with high SCORE 10-y CVD mortality risk. Ten-year CVD mortality risks were calculated using the SCORE European low-risk equation in 1,047 participants younger than 65 y without cardiovascular disease and/or symptoms at baseline.

**Figure 6:**
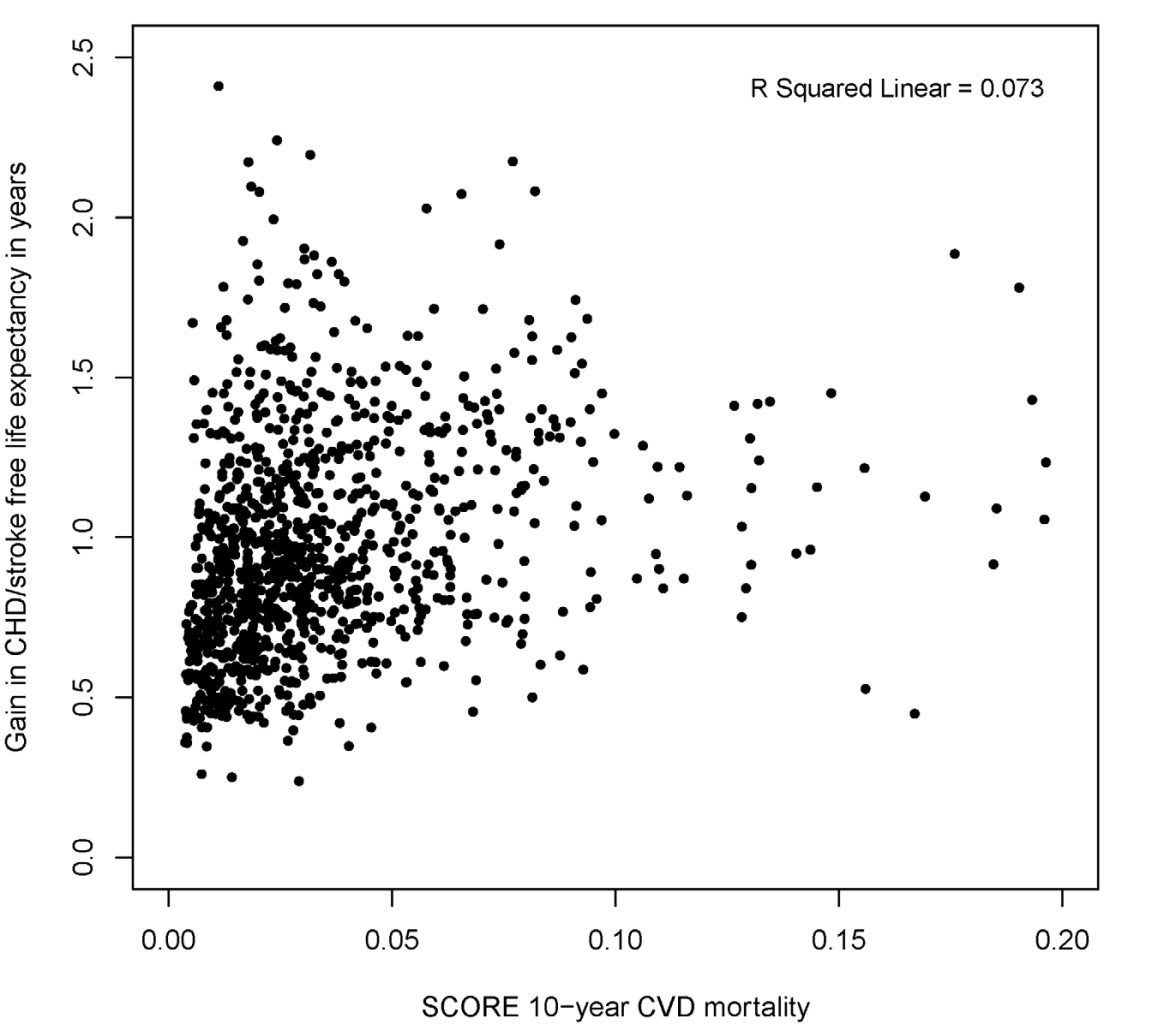
Distribution of gains in CHD/stroke-free life expectancy according to SCORE 10-y total CVD mortality risk (percent). Note that many individuals with a low SCORE 10-y CVD mortality risk achieved similar or higher gains than those with high SCORE 10-y CVD mortality risk. Ten-year CVD mortality risks were calculated using the SCORE European low-risk equation in 1,047 participants younger than 65 y without cardiovascular disease and/or symptoms at baseline. SCORE = Systematic COronary Risk Evaluation.

We compared the low-risk region SCORE charts with the predicted gain in life expectancy with statin therapy (Figure 2). These charts demonstrate that the 10-y total CVD mortality risk is highest for elderly smoking individuals with otherwise high risk factor levels, suggesting that these individuals would benefit most from statin therapy. Figures 3 and 4, however, demonstrate that the lifetime benefits of statin therapy are highest for young non-smoking individuals with high systolic blood pressure and cholesterol levels. For example, a 55-y-old non-smoking female at a 10-y CVD mortality risk of 1% could achieve a similar gain in total life expectancy with statin therapy as a 65-y-old smoking male at a risk of 26%. Figures 5 and 6 plot SCORE risk estimations versus gains in total and CHD/stroke-free life expectancy. These plots demonstrate that many individuals with low SCORE values achieved similar or larger gains than those with high SCORE values. In Figure 5, 19% and in Figure 6, 25% of the participants with a SCORE below 0.05 had benefits greater than or equal to the gains observed in 50% of the population with a SCORE of 0.05 or more.

## Discussion

In this modeling study, we found that in 2,428 asymptomatic participants, statin therapy resulted in robust, small gains in total life expectancy and somewhat larger gains in CHD/stroke-free life expectancy. The expected benefit of statin therapy was determined by a number of baseline variables. From these variables, we constructed a web-based calculator and color charts. Once the underlying model has been independently validated, these tools can be used for communication of the expected lifetime benefits of statin therapy in persons aged 55 y and older. Inconsistencies occurred between the predicted benefits and what can be expected from the currently recommended 10-y CVD risk assessment. These inconsistencies were predominantly caused by age, which acts on lifetime benefits in the opposite direction as its effect on 10-y CVD risk. Individuals at low 10-y CVD risk may achieve a similar or even larger gain in total and CHD/stroke-free life expectancy as those at high 10-y risk.

Most decision tools for CVD prevention in asymptomatic individuals predict the individual's risk over a time period ranging from 5 to 10 y without calculating potential treatment benefits [1]. If treatment benefits are presented, they are usually calculated as absolute risk reductions without taking into account competing risks [21]–[25]. Two decision tools for making choices on statin therapy were based on Markov models predicting lifetime outcomes with and without statin therapy [26],[27]. The underlying decision models used data from multiple sources for estimating CVD events, and age- and sex-specific life tables for competing death probabilities, which are not necessarily compatible [28]. In contrast, we used event probability estimations from one data source. Furthermore, we modeled the occurrence of stroke events separately from CHD events. Statin therapy has a different effect on strokes [3], and ignoring this effect would lead to incomplete estimation and communication of treatment benefits.

Despite these strengths, our results must be interpreted in the light of some limitations. One limitation is that the RISC model was used to extrapolate 5-y predictions to a lifetime horizon, which may be very sensitive to the method chosen [29]. The RISC model extends cumulative incidence functions by updating age and risk factor levels using 5-y time intervals. Secular trends in risk factor levels were modeled across the age span using cross-sectional data, and thus potential chronological and cohort effects were not taken into account. We evaluated the validity of these extrapolations with subsequently available Rotterdam Study data not used in developing the RISC model and found that the deviations were generally limited. Developing predictions on longer follow-up data, e.g., 30 y, would allow for a more comprehensive evaluation of long-term validity [30]. However, this approach is also questioned, given chronological changes in CVD event rates and associated risk factors [31],[32], which are less likely to affect validity if more recent and thus shorter follow-up data are used [33]. We did not evaluate the model's performance on predicting outcomes at the individual level (discrimination) and group level (calibration) using external data. This would be necessary to investigate to what extent the personalized predictions are transportable to other settings and geographical sites, but is beyond the scope of this study.

A second limitation is that the relative risk reducing effect of statin therapy was kept constant over age and various risk factor levels. Although a number of observational studies [34] found that the protective effect of cholesterol lowering on CVD events decreases in individuals aged 70 to 89 y, this was not confirmed by experimental research [20],[21]. Meta-analyses of statin trials demonstrate that effects on cardiovascular events are fairly independent of various risk factor levels [3],[35]. These trials, however, predominantly included participants with elevated risk factor levels. In the Rotterdam Study, individuals with normal risk factor levels were also included, and it is not known whether the relative risk reduction will be different for these individuals with normal levels. Thus, we cannot exclude the possibility of a small overestimation of the statin therapy effect in those with normal risk levels.

A third limitation is that, although we did account for baseline statin use, we did not take into account initiation of statin therapy during follow-up. Omitting this information could lead to an underestimation of the effect of statin therapy. However, in the 1990s, mass screening for dyslipidemia was not advocated, and statins were prescribed only to patients with a history of CVD or with persistent severe dyslipidemia after dietary intervention [36]. Follow-up examinations of the Rotterdam Study population in 1997 revealed that statin use was quite limited [37]. Thus, underestimation of statin effects due to treatment drop-ins will be small.

A fourth limitation is that the RISC model's outcomes did not perfectly match with all the outcomes as evaluated within statin trials. Therefore, we were not able to model a statin effect on total stroke events, and solely modeled an effect on first ischemic and unspecified stroke. However, these stroke subtypes contribute to 92% of all first stroke events in the Rotterdam Study [38]. In addition, we did not model a direct statin effect on CVD mortality by causes other than myocardial infarction and stroke. Although a reduction in sudden cardiac death—a major component of CVD mortality—is observed in symptomatic patients treated with statins, the effect for participants without manifest CVD seems negligible [39]. Nevertheless, we cannot exclude a small underestimation of benefits due to these modeling choices.

A final limitation is that our results may not be generalizable to other populations. The RISC model's output on cardiovascular mortality was most compatible with a population resembling inhabitants of a low CVD risk region. This finding confirms results from another cohort study [16], suggesting that cardiovascular mortality in Dutch individuals is most similar to predictions from the low risk region SCORE equation. In addition, the generalizability of our results also depends on the competing rate of mortality due to other diseases. Our estimations of remaining life expectancy for females and males at the ages of 60, 65, and 80 y, however, reasonably match estimations for low CVD risk countries as projected by the Organisation for Economic Co-operation and Development [40]. Thus, the web-based calculator and color charts should be used with caution in individuals from regions with higher CVD risk.

The competing mortality risks from other CVD and non-CVD causes of death, which were not affected by statin therapy, sometimes resulted in counterintuitive lifetime outcomes. For example, age is the most important factor increasing both the yearly probability of CHD and stroke events, and the fatality of these events. Thus, age is expected to increase the health benefit of statin therapy. However, in the Rotterdam Study, age is even more strongly associated with an increase in yearly mortality from other causes of death [9]. Subsequently, changes with statin therapy in lifetime outcomes were smaller with increasing age, because prevented CHD and stroke events were also increasingly substituted by other fatal events.

Although the average gain in total life expectancy with statin therapy may seem small, it is larger than that calculated for some other preventive interventions targeted at the general population [29]. One should recognize that gains were much larger in particular participants, and were averaged out by participants who never experienced CVD. It should also be acknowledged that with the benefits of statin therapy, the costs, side effects, and disutility of daily pill use are likely to be acceptable across various age groups and risk levels, especially in a “low statin cost era” [41],[42]. In addition, we observed that gains in CHD/stroke-free life expectancy were generally larger than those in total life expectancy. Two phenomena can explain this observation. First, a large proportion of the CHD and stroke events were not fatal. Gains in CHD/stroke-free life expectancy are mainly driven by statin effects on non-fatal CHD and stroke event rates, while gains in total life expectancy are driven by effects on CHD and stroke death rates. Second, individuals in whom fatal CHD and stroke events are avoided are also likely to be at elevated risk for death by other causes. Our finding of a smaller effect of statin therapy on total life expectancy than on CHD/stroke-free life expectancy is in agreement with the results from statin trials, in which generally only modest effects are demonstrated for crude total mortality risks, while effects on crude CHD and stroke incidence risks are more pronounced [3].

Currently, statin therapy choices are based on short-term CVD risk without statin therapy and the expected risk reduction with statin therapy over the same time period. We converted survival benefits with statin therapy into total life expectancy and CHD/stroke-free life expectancy.

We believe that the prediction of statin therapy effects on (disease-free) life expectancy can be complementary to the 10-y CVD risk assessment in two ways. First, instead of regarding a fixed time point, i.e., 10 y, the benefit of statin therapy considering the entire survival curve can be communicated by primary care physicians. Second, the benefit of statin therapy is calculated taking into account competing mortality risks. The potential value of personalizing the gain in total and CHD/stroke-free life expectancy with statin therapy is best illustrated by Figures 5 and 6. A substantial number of individuals with 10-y total CVD mortality risk lower than 5%, for whom statin therapy is generally not recommended according to current European Society of Cardiology guidelines, may benefit to the same extent as individuals with a high risk. A similar pattern will apply to predictions based on other CVD risk models, such as risk scores based on the Framingham Study [43],[44], because these use the same risk factors, with effects pointing in equal directions.

When making decisions on statin therapy, the fact that the benefit in life expectancy diminishes with advancing age may be considered by physicians, especially in the elderly. If independently validated, physicians could use the web-based calculator and color charts to frame survival outcomes in different ways and to discuss them with the patient in light of the expected duration of statin use. The longer the life expectancy, and therefore the expected duration of statin use, the higher the costs and possibility of adverse effects. Besides the costs averted by CVD prevention, these important outcomes could influence the decision, but were not taken into account in our analysis. In addition, it should be acknowledged that the calculated differences in the personalized lifetime outcomes may vary across different clinical settings and are subject to the parameter uncertainty in the underlying decision model. These caveats would need to be discussed with patients when they are informed about the benefits of statin therapy.

In conclusion, we demonstrated that life expectancy benefits with statin therapy can be predicted using an individual's risk factor profile. The predicted gains in life expectancy we found are generally small. If the underlying model is validated in an independent cohort, the developed tools may be useful in discussing with patients their individual expected outcomes with statin therapy. Ideally, communication of personalized outcomes will ultimately result in better clinical outcomes. Improved understanding of potential gains will, however, not necessarily go hand in hand with an improvement in clinical outcomes, because patients could be less likely to choose statin therapy when more information on benefits is provided [45]. In addition to an external validation of our predictions, personalized estimates for costs and side effects of statin therapy should be included in future research. Finally, the impact of communicating life expectancy benefits on satisfaction, behavioral, and clinical outcome measures should be studied.

## Supporting Information

Figure S1 **Comparison of RISC model 10-y CVD mortality and SCORE low-risk region 10-y CVD mortality.** Ten-year CVD mortality risks were calculated by tenth of RISC model 10-y CVD mortality risk for 1,047 participants younger than 65 y without cardiovascular disease and/or symptoms at baseline.
(TIFF)

Figure S2 **Comparison of RISC model 10-y CVD mortality and SCORE high-risk region 10-y CVD mortality.** Ten-year CVD mortality risks were calculated by tenth of RISC model 10-y CVD mortality risk for 1,047 participants younger than 65 y without cardiovascular disease and/or symptoms at baseline.
(TIFF)

Figure S3 **Comparison of RISC model 10-y CVD mortality and Dutch recalibrated SCORE 10-y CVD mortality.** Ten-year CVD mortality risks were calculated by tenth of RISC model 10-y CVD mortality risk for 1,047 participants younger than 65 y without cardiovascular disease and/or symptoms at baseline.
(TIFF)

Table S1 **RISC model input parameters.**
(DOCX)

Table S2 **RISC model predictions for lifetime CHD/stroke incidence, CHD/stroke mortality, and total CVD mortality for four risk profiles.**
(DOCX)

Text S1 **Appendix.**
(DOC)

## Abbreviations

CHD: coronary heart disease
CVD: cardiovascular disease
HDL: high-density lipoprotein
ICD-10: International Classification of Diseases, 10th edition
RISC model: Rotterdam Ischemic Heart Disease & Stroke Computer Simulation Model
SCORE: Systematic Coronary Risk Evaluation
SD: standard deviation
95% CI: 95% confidence interval
