# Supplementary figures and images for: Personalized Prediction of Lifetime Benefits with Statin Therapy for Asymptomatic Individuals: A Modeling Study
Source: PLoS Med. 2012 Dec 27;9(12):e1001361. doi: 10.1371/journal.pmed.1001361 (PMC3531501; doi:10.1371/journal.pmed.1001361)

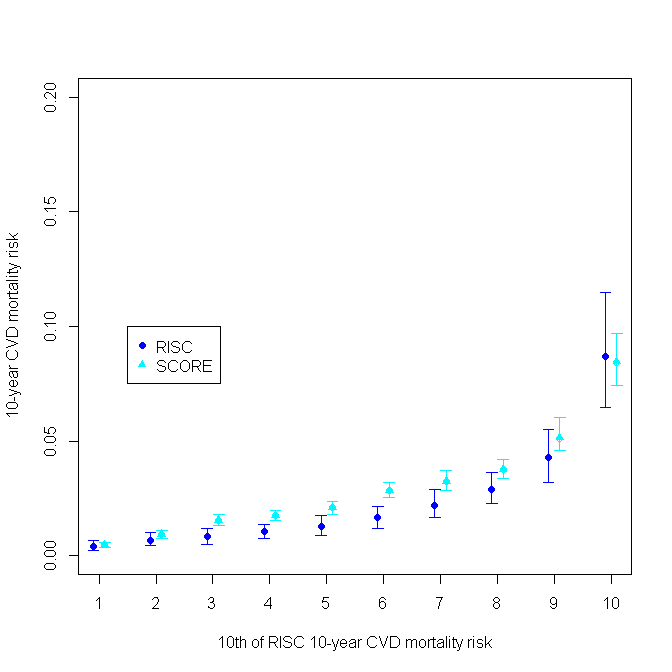

Supplement: Figure S1 — Comparison of RISC model 10-y CVD mortality and SCORE low-risk region 10-y CVD mortality. Ten-year CVD mortality risks were calculated by tenth of RISC model 10-y CVD mortality risk for 1,047 participants younger than 65 y without cardiovascular disease and/or symptoms at baseline. (TIFF) [file pmed.1001361.s001.tiff]

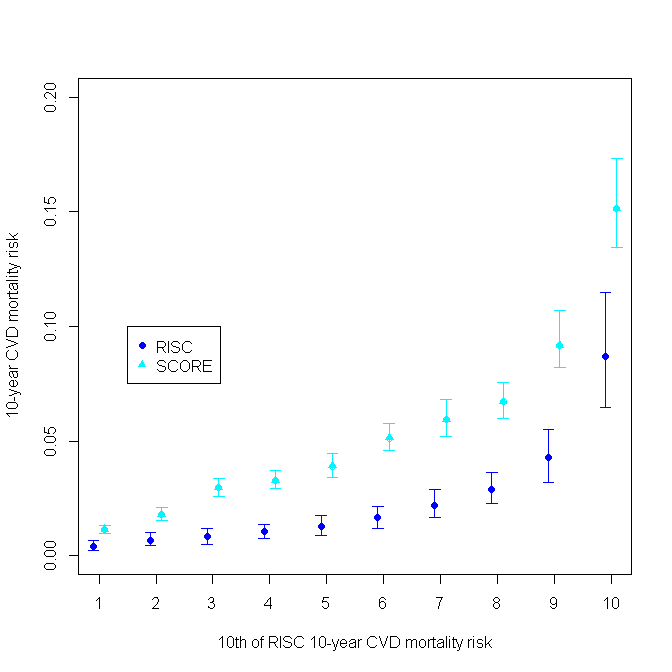

Supplement: Figure S2 — Comparison of RISC model 10-y CVD mortality and SCORE high-risk region 10-y CVD mortality. Ten-year CVD mortality risks were calculated by tenth of RISC model 10-y CVD mortality risk for 1,047 participants younger than 65 y without cardiovascular disease and/or symptoms at baseline. (TIFF) [file pmed.1001361.s002.tiff]

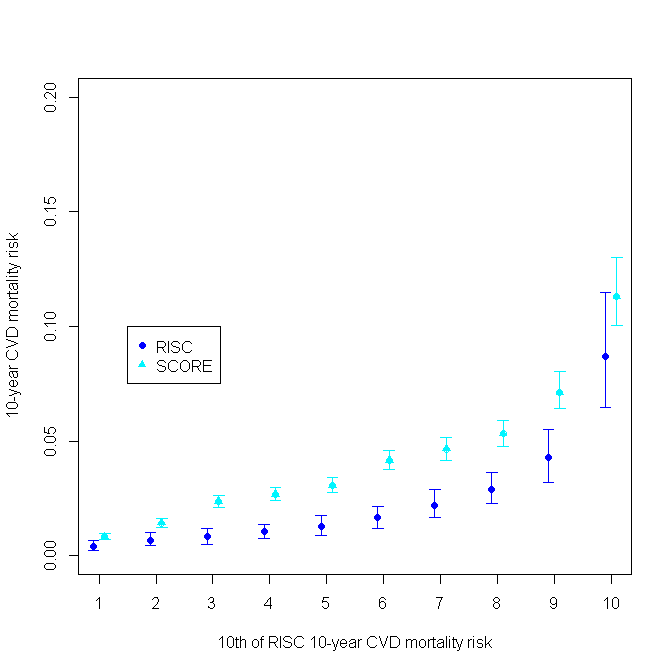

Supplement: Figure S3 — Comparison of RISC model 10-y CVD mortality and Dutch recalibrated SCORE 10-y CVD mortality. Ten-year CVD mortality risks were calculated by tenth of RISC model 10-y CVD mortality risk for 1,047 participants younger than 65 y without cardiovascular disease and/or symptoms at baseline. (TIFF) [file pmed.1001361.s003.tiff]
