# Supplementary material for: Personalized Prediction of Lifetime Benefits with Statin Therapy for Asymptomatic Individuals: A Modeling Study
Source: PLoS Med. 2012 Dec 27;9(12):e1001361. doi: 10.1371/journal.pmed.1001361 (PMC3531501; doi:10.1371/journal.pmed.1001361)
Supplement: Table S1 — RISC model input parameters. (DOCX) [file pmed.1001361.s004.docx]

**Table S1.** **RISC Model input parameters**

| Outcomes | Cox-regression equations* |
| --- | --- |
| CHD | Hazard function = baseline cumulative hazard x EXP(b1*male+b2*age+b3*age*age+b4*diabetes*glucose+b5*TC+b6*HDL+b7*PP+b8*PP*male+b9*angina pectoris+b10*ABI+b11*ABI*ABI+b12*smoking+b13*famhistMI+b14*CVD+b15*creat-mean linear predictor) |
| Stroke | Hazard function = baseline cumulative hazard x EXP(b1*male+b2*age+b3*hypertension+b4*hypertension*age+b5*SBP+b6*smoking+b7*famhistMI+b8*TIA+b9*CVD+  b10*CVD*male+b11*AF+b12*ABI-mean linear predictor) |
| 6-months CHD event mortality | Hazard function = baseline cumulative hazard x  EXP(b1*age+b2*diabetes*glucose+b3*hypertension+b4*hypertension*age+b5*creat-mean linear predictor) |
| 6-months Stroke event mortality | Hazard function = baseline cumulative hazard x  EXP(b1*age+b2*smoking+b3*famhistCVD+b4*ABI+b5*TC+b6*creat+b7*HDL+b8*ABI*ABI+b9*age*HDL-mean linear predictor) |
| other CVD mortality | Hazard function = baseline cumulative hazard x  EXP(b1*age+b2*male+b3*diabetes+b4*HDL*CVD+b5*hypertension+b6*hypertension*age+b7*smoking+b8*CVD+b9*ABI+b10*ABI*ABI+b11*AF+b12*AF*male+b13*age*AF+b14*male*CVD+b15*CVD*TC*TC-mean linear predictor) |
| Non-CVD mortality | Hazard function = baseline cumulative hazard x  EXP(b1*male+b2*age+b3*glucose+b4*TC+b5*smoking+b6*smoking*age+b7*BMI+b8*BMI*age+b9*WHR+b10*WHR*age+b11*WHR*CVD+b12* famhistCVD+b13* famhistCVD*age+b14*ABI+b15*ABI*age+b16*CVD-mean linear predictor) |
| Statin Therapy Effects | Odds Ratio (95% Confidence interval) |
| CHD risk reduction | 0.70 (0.61 – 0.81) |
| Stroke risk reduction | 0.81 (0.71 – 0.93) |

*Beta coefficients were drawn from a table comprising estimated beta coefficients from Cox regression equations developed in 100 bootstrapped datasets.

ABI = ankle-brachial index. AF = atrial fibrillation. BMI = body mass index. CHD = coronary heart disease. Creat = creatinine. CVD = cardiovascular disease. FamhistMI = family history of myocardial infarction. FamhistCVD = family history of cardiovascular disease. HDL = high-density lipoprotein. PP = pulse pressure. SBP = systolic blood pressure. TC = total cholesterol. TIA = transient ischaemic attack. WHR = waist-to-hip ratio.
