# Supplementary material for: Personalized Prediction of Lifetime Benefits with Statin Therapy for Asymptomatic Individuals: A Modeling Study
Source: PLoS Med. 2012 Dec 27;9(12):e1001361. doi: 10.1371/journal.pmed.1001361 (PMC3531501; doi:10.1371/journal.pmed.1001361)
Supplement: Table S2 — RISC model predictions for lifetime CHD/stroke incidence, CHD/stroke mortality, and total CVD mortality for four risk profiles. (DOCX) [file pmed.1001361.s005.docx]

**Table S2. RISC Model predictions for lifetime CHD/stroke incidence, CHD/stroke mortality, and total CVD mortality for four risk profiles**

| **Risk Profile** | Lifetime CHD/stroke incidence | | Lifetime CHD/  stroke mortality | | Lifetime total CVD mortality | |
| --- | --- | --- | --- | --- | --- | --- |
|  | Statin - | Statin + | Statin - | Statin + | Statin - | Statin + |
| 55 yr old, non-smoking ♀, blood pressure 140/80 mm Hg, hypertension +, total cholesterol 6.0 mmol/L, HDL cholesterol 1.5 mmol/L, diabetes -, glucose 6.0 mmol/L, BMI 25.0, WHR 0.80, creatinine 80 μmol/L | 42% | 34% | 17% | 13% | 43% | 42% |
| 65 yr old, smoking ♂, blood pressure 130/70 mm Hg, hypertension +, total cholesterol 7.0 mmol/L, HDL cholesterol 1.0 mmol/L, diabetes +, glucose 6.0 mmol/L, BMI 30.0, WHR 1.06, creatinine 90 μmol/L | 52% | 43% | 19% | 13% | 52% | 49% |
| 55 yr old, non-smoking ♂, blood pressure 140/75 mm Hg, hypertension +, total cholesterol 7.0 mmol/L, HDL 1.3 mmol/L, diabetes -, glucose 6.5 mmol/L, BMI 27.0, WHR 1.00, creatinine 80 μmol/L | 55% | 46% | 18% | 13% | 48% | 45% |
| 75 yr old, smoking ♂, blood pressure 120/80 mm Hg, hypertension +, total cholesterol 4.5 mmol/L, HDL 1.0 mmol/L, diabetes +, glucose 6.0 mmol/L, BMI 21.0, WHR 1.00, creatinine 90 μmol/L | 25% | 19% | 10% | 7% | 31% | 29% |

Hypertension is defined as either reporting use of antihypertensive medication or having a systolic blood pressure ≥ 160 mmHg or a diastolic blood pressure ≥ 95 mmHg; diabetes is defined as either reporting use of antidiabetic medication or having a serum glucose level ≥ 11.0 mmol/L.

BMI = body mass index. CHD = coronary heart disease. CVD = cardiovascular disease. HDL = high-density lipoprotein. WHR = waist-to-hip ratio.
