# Supplementary material for: Personalized Prediction of Lifetime Benefits with Statin Therapy for Asymptomatic Individuals: A Modeling Study
Source: PLoS Med. 2012 Dec 27;9(12):e1001361. doi: 10.1371/journal.pmed.1001361 (PMC3531501; doi:10.1371/journal.pmed.1001361)
Supplement: Text S1 — Appendix. (DOC) [file pmed.1001361.s006.doc]

### Text S1. Appendix.

In this Appendix, we provide additional information on the Rotterdam Study, the RISC model and performed analyses.

**Description of the Rotterdam Study**

The Rotterdam Study is a population-based cohort study of subjects aged 55 years or older living in the well-defined Ommoord district in the city of Rotterdam, the Netherlands. The study targets cardiovascular, endocrine, hepatic, neurological, ophthalmic, psychiatric and respiratory diseases in the elderly. The Rotterdam Study has been approved by the Medical Ethics Committee of the Erasmus Medical Center and by the review board of The Netherlands Ministry of Health, Welfare and Sports [1].

The cohort used for this study had baseline examinations between July 1989 and September 1993. These examinations comprised extensive clinical examinations at the research center and an interview at home including questions on current health status, history of cardiovascular diseases, medication use and cardiovascular risk factors. Smoking behaviour was categorized into current, former or never smoking. Former smoking was defined as abstinence of at least two years of smoking. Participants were asked to fast for 12 hours before the clinical examinations. For systolic and diastolic blood pressure, the average of two consecutive measurements was used. Blood pressure was measured with a random-zero sphygmomanometer at the right brachial artery in sitting position after a 5 minutes rest. Hypertension was defined as either reported use of antihypertensive medication, or having a systolic blood pressure ≥ 160 mmHg or diastolic blood pressure ≥ 95 mmHg. Serum total cholesterol was determined by an automated enzymatic procedure using the Roche CHOD-PAP reagent agent and serum high density lipoprotein (HDL)-cholesterol was measured with the Roche HDL cholesterol assay using PEG-modified enzymes and dextran sulphate. Diabetes mellitus was defined as either reported use of antidiabetic medication or a serum glucose level ≥ 11.0 mmol/L. Twelve-lead resting electrocardiograms were computer-analyzed by the MEANS program [2]. Angina pectoris was assessed using the Rose questionnaire [3]. The ankle-brachial index was calculated as the ratio of the systolic blood pressure of the posterior tibial artery, measured by an 8 MHz continuous wave Doppler probe and a random-zero sphygmomanometer, to the systolic blood pressure at the arm. The lowest ABI in either side was used and the left and right sided readings combined for analysis.

Outcome data were continuously collected from general practitioners and hospital discharge reports. All events were classified independently by two research physicians. If disagreements occurred, a consensus was reached by a meeting. Finally, all events were verified by a cardiologist or neurologist affiliated with the study. In cases of unresolved discrepancy, the judgment by the expert was considered definite.

**RISC Model**

The RISC model consists of six health states: Well, Coronary Heart Disease (CHD), Stroke, CHD & Stroke, Cardiovascular Death and Non-cardiovascular Death (see Figure 1). Probabilities for the transitions between the six health states were based on six multivariable Cox regression equations. The development of these equations was described in a previous article on the RISC model [4]. The first equation estimated the cumulative hazard from the Well state to the CHD state and from the Stroke state to the CHD & Stroke state. The second equation estimated the cumulative hazard from the Well state to the Stroke state and from the CHD state to the CHD & Stroke state. In developing these models, censoring was performed for an incident stroke and CHD respectively. In both equations, previous CHD and/or stroke were included as a co-variable. The third and fourth equations estimated the 6-months case-fatality after a CHD and stroke event respectively. Six-month cardiovascular mortality as defined above was used as outcome for the case-fatality rates of these events. The probability of dying from a fourth CHD event and third stroke event was assumed to be 100%. The fifth and sixth Cox regression equations estimated the cumulative hazards of the remaining CVD mortality (other than CHD or stroke within 6 months) and non-CVD mortality (see Table S1 for equations). For extrapolation to a lifelong follow-up, follow-up time was divided into 5-year intervals and a cycle length of one year was used. The first 5 years, baseline values of co-variables were used together with the one-year cumulative hazards from the Cox models for each cycle. For the remaining follow-up, the same baseline one-year cumulative hazards were used, but values of the co-variables were updated every 5 years by using multiple linear regression for the continuous variables systolic blood pressure, diastolic blood pressure, total cholesterol, HDL-cholesterol, plasma glucose, ankle-brachial index, body mass index, waist-to-hip ratio, and serum creatinine, and logistic regression for the dichotomized variables atrial fibrillation, angina pectoris, and diabetes. Age and sex were used as independent variables in these regression equations to take into account the secular changes in risk factor levels due to aging for males and females separately. From the outcomes of the logistic regression equations regarding dichotomized variables, binomial distributions were created. Every 5 year period presence (1 or 0) of the dichotomized variables was derived from these distributions. The updating of the co-variables each 5-year period required the use of tracker variables. One-year cumulative hazards of CHD, stroke, other CVD mortality, and non-CVD mortality were weighted for their total cumulative hazards to take into account the competing risks for occurrence of a first event within each cycle. For each transition, the weighted one-year cumulative hazard was converted to a one-year cumulative incidence by exponentiation. If constant cause-specific hazards are assumed for the events, the cumulative incidence for each event will be estimated correctly [5]. This is a reasonable assumption given the cycle length of one year. The effect of statin therapy on the transitions from the Well state to the CHD state or the Stroke state was modelled by multiplying the odds ratio of statin therapy after converting probabilities to the odds scale. Occurrences of events and duration in each health state were monitored using Monte Carlo tracker variables.

**Statistical Analyses**

For the two main outcomes, life expectancy and CHD/stroke free life expectancy, extended linear models with main effects of all variables, quadratic terms of all continuous variables, and interaction terms with statin therapy, age and sex were constructed. An unstructured covariance matrix was selected for both outcomes. Parameters were removed on the basis of improvement in the Akaike’s Information Criterion (AIC), using the R function stepAIC, which calculates the log-likelihood penalized for the number of parameters and subjects used. The best candidate model is the one with the minimum AIC value. Final models were fitted with the restricted maximum likelihood method. A web-based calculator was constructed in using the equations derived from the models. For modelling CVD event and mortality risks, generalized linear models were fitted with the number of tracked events and non-events per 20,000 runs as dependent variable using a logit link function and binomial distribution type. To ensure face validity, all predictors selected for the main outcome measures were used as independent variables in the generalized linear models predicting CVD event and mortality risks.

### References

1. Hofman A, van Duijn CM, Franco OH, Ikram MA, Janssen HL, et al. (2011) The Rotterdam Study: 2012 objectives and design update. Eur J Epidemiol 26: 657-686.

2. van Bemmel JH, Kors JA, van Herpen G (1990) Methodology of the modular ECG analysis system MEANS. Methods Inf Med 29: 346-353.

3. Kuller LH, Shemanski L, Psaty BM, Borhani NO, Gardin J, et al. (1995) Subclinical disease as an independent risk factor for cardiovascular disease. Circulation 92: 720-726.

4. Nijhuis RL, Stijnen T, Peeters A, Witteman JC, Hofman A, et al. (2006) Apparent and internal validity of a Monte Carlo-Markov model for cardiovascular disease in a cohort follow-up study. Med Decis Making 26: 134-144.

5. Koller MT, Raatz H, Steyerberg EW, Wolbers M (2011) Competing risks and the clinical community: irrelevance or ignorance? Stat Med.
